# Identification and verification of ferroptosis-related genes in gastric intestinal metaplasia

**DOI:** 10.3389/fgene.2023.1152414

**Published:** 2023-04-18

**Authors:** Biao Song, Tingting Li, Yi Zhang, Qi Yang, Bei Pei, Yun Liu, Jieyu Wang, Gang Dong, Qin Sun, Shanshan Fan, Xuejun Li

**Affiliations:** ^1^ The Graduated School, Anhui University of Traditional Chinese Medicine, Hefei, China; ^2^ Department of Gastroenterology, The Second Affiliated Hospital of Anhui University of Traditional Chinese Medicine, Hefei, China; ^3^ Ningbo University of Technology, Ningbo, China

**Keywords:** intestinal metaplasia, ferroptosis, bioinformatics, immune landscape, diagnosis

## Abstract

**Background:** Gastric intestinal metaplasia (IM) is the key link of gastric precancerous lesions. Ferroptosis is a novel form of programmed cell death. However, its impact on IM is unclear. The focus of this study is to identify and verify ferroptosis-related genes (FRGs) that may be involved in IM by bioinformatics analysis.

**Materials and methods:** Differentially expressed genes (DEGs) were obtained from microarray dataset GSE60427 and GSE78523 downloaded from Gene Expression Omnibus (GEO) database. Differentially expressed ferroptosis-related genes (DEFRGs) were obtained from overlapping genes of DEGs and FRGs got from FerrDb. DAVID database was used for functional enrichment analysis. Protein-protein interaction (PPI) analysis and Cytoscape software were used to screen hub gene. In addition, we built a receiver operating characteristic (ROC) curve and verified the relative mRNA expression by quantitative reverse transcription-polymerase chain reaction (qRT-PCR). Finally, the CIBERSORT algorithm was used to analyze the immune infiltration in IM.

**Results:** First, a total of 17 DEFRGs were identified. Second, a gene module identified by Cytoscape software was considered as hub gene: PTGS2, HMOX1, IFNG, and NOS2. Third, ROC analysis showed that HMOX1 and NOS2 had good diagnostic characteristics. qRT-PCR experiments confirmed the differential expression of HMOX1 in IM and normal gastric tissues. Finally, immunoassay showed that the proportion of T cells regulatory (Tregs) and macrophages M0 in IM was relatively higher, while the proportion of T cells CD4 memory activated and dendritic cells activated was lower.

**Conclusion:** We found significant associations between FRGs and IM, and HMOX1 may be diagnostic biomarkers and therapeutic targets for IM. These results may enhance our understanding of IM and may contribute to its treatment.

## 1 Introduction

Gastric cancer (GC) is one of the most common cancer and a cause of death worldwide. According to statistics from the World Health Organization (WHO) in 2020 (Sung et al., 2021), the incidence of GC ranks fifth in all kinds of cancer in the world, and it is the fourth leading cause of cancer-related death. The development of GC is a gradual process, and its histological progression was first described by Correa and Piazuelo (2012). GC’s histological progression called Correa cascade reaction of gastric carcinogenesis, shows the histological pathway from normal gastric mucosa to gastric cancer (normal gastric mucosa → non-atrophic gastritis → atrophic gastritis → intestinal metaplasia (IM) → dysplasia → gastric cancer). IM is the key link of gastric precancerous lesions and is regarded as an important inducer for the development of intestinal-type GC (Kinoshita et al., 2017; Shao et al., 2018). Gastric intestinal metaplasia (GIM) defined as that the gastric columnar cells are replaced by intestinal morphology cells, is characterized by the mucin-containing goblet, Paneth and absorptive cells, causing normal gastric mucosal epithelium and surrounding glands replaced by intestinal epithelium and glands (Shah et al., 2020a). Based on a comprehensive systematic review, Gastric IM is associated with the baseline gastric cancer risk (annually 0.16%) (Gawron et al., 2020). With the development of chronic mucosal inflammation, IM is considered as a response to injury, but we know little about its mechanism (Goldenring and Mills, 2022). Now, there is no specific treatment for IM. Regular surveillance in high-risk patients and the prevention of IM is one of the main management methods recommended by the guidelines (Shah et al., 2020b). Therefore, a more comprehensive understanding of IM and proper monitoring of IM patients may improve GC-related morbidity and mortality.

Ferroptosis is an iron-dependent form of regulated cell death driven by the lethal accumulation of lipid peroxidation (Dixon et al., 2012). Ferroptosis plays a role in the development and progression of many diseases, including cancer, necroinflammatory disorders, and many organ damages and degenerative changes (Jiang et al., 2021). Previous studies have revealed the important role of ferroptosis in GC (Zhang et al., 2020; Liu et al., 2021; Gu et al., 2022). Ferroptosis plays an important regulatory role in the development of malignant tumors such as proliferation, invasion and metastasis of GC (Guan et al., 2022; Lu et al., 2022). However, no attention has been reported to the role of ferroptosis in gastric precancerous lesions, let alone in IM. Therefore, the purpose of this study is to explore the role of ferroptosis-related genes (FRGs) in IM through bioinformatics analysis, and to analyze and verify the accuracy of related gene diagnosis models as IM biomarkers.

## 2 Materials and methods

### 2.1 Data collection

**FIGURE 1:**
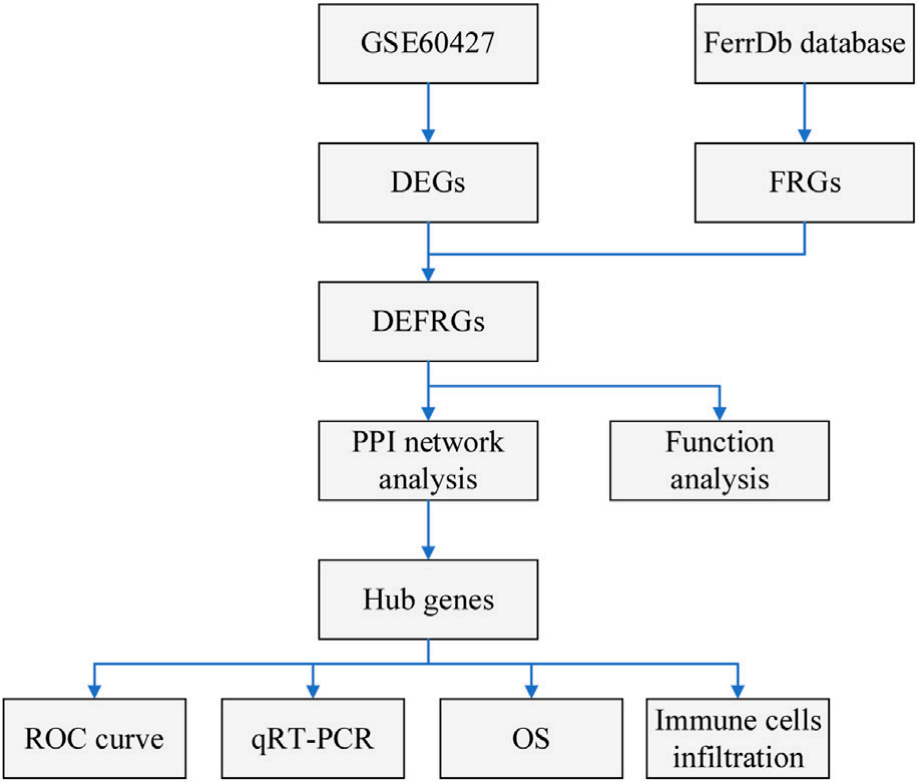
Flowchart of the study.

Figure 1 shows the flow chart of our study. The gene expression data of IM and normal samples were obtained from Gene Expression Omnibus (GEO) database (http://www.ncbi.nlm.nih.gov/geo/). GSE60427 contained 16 normal samples and 8 IM samples, and was used as a training set for subsequent analysis. GSE78523 contained 15 normal samples and 30 IM samples, and was used to verify the expression of the marker genes. In addition, the FRGs used in this study were derived from the FerrDb database (Zhou and Bao, 2020) (http://www.zhounan.org/ferrdb).

### 2.2 Identification of differently expressed ferroptosis-related genes

The “limma” package in R software was used to screen differentially expressed genes (DEGs) between IM and normal subjects. The screening criterion was adjusted *p* < 0.05 and |log2FC| ≥ 1. We selected overlapping DEGs of FRGs as differently expressed ferroptosis-related genes (DEFRGs). “volcano” and “heatmap” R packages were used to visualize differential genes.

### 2.3 Functional enrichment analysis

The DAVID database (https://david.ncifcrf.gov/) was used for GO biological processes analysis, including molecular function (MF), biological process (BP) and cellular component (CC), and KEGG pathway enrichment analysis of the above differentially expressed genes, with *p* < 0.05 as the screening threshold.

### 2.4 Protein-protein interaction network and identification of hub genes

The STRING online website (https://string-db.org/) was used to build a DEFRGs interaction network, with moderate confidence (0.4) as the required minimum interaction score. Then the results of STRING were imported into Cytoscape, and the hub genes were extracted by MCODE plug-in and cytoHubba plug-in.

### 2.5 Collection of tissue specimens

We collected 5 GIM and 5 healthy gastric tissue samples from patients in the Second Affiliated Hospital of Anhui University of Chinese Medicine. All patients understood the nature of the study and signed the informed consent form before participating in the study. The Ethics Committee of the Second Affiliated Hospital of Anhui University of Chinese Medicine reviewed and approved this study.

### 2.6 Real-time quantitative reverse transcription PCR

Total RNA was extracted from each sample by using Trizol reagent (Life technogies, Shanghai, China). Reverse transcription was performed with gDNA Eraser kit (TaKaRa, Beijing, China). qRT-PCR primers were provided by Sangon Biotech (China). The primer sequences were described by Table 1. *β*-Actin gene was used as an internal control. 2^−ΔΔCt^ comparison method was used for relative quantification.

**TABLE 1:** PCR primer sequences.

Gene	Forward primer	Reverse primer
	(5′→3′)	(5′→3′)
*β*-Actin	CCC​TGG​AGA​AGA​GCT​ACG​AG	GGA​AGG​AAG​GCT​GGA​AGA​GT
HMOX1	TCT​CTG​GAA​AGG​AGG​AAG​GA	AGG​AAC​TGA​GGA​TGC​TGA​AG
NOS2	TGT​AGC​GAG​TCG​AAA​ACT​GA	GGG​TAA​GGA​CAG​TCA​AAC​CA

### 2.7 Immune infiltration analysis

To evaluate the function of immune microenvironment in IM formation, we used CIBERSORT algorithm (Newman et al., 2015) to quantify the relative abundance of 22 types of infiltrating immune cells in IM and normal samples. Wilcoxon rank sum test was used to compare the difference of immune infiltration between IM patients and normal samples. Spearman correlation analysis was performed to show the association between hub gene and differential infiltrating immune cells.

## 3 Results

### 3.1 Detection of DEFRGs

**FIGURE 2:**
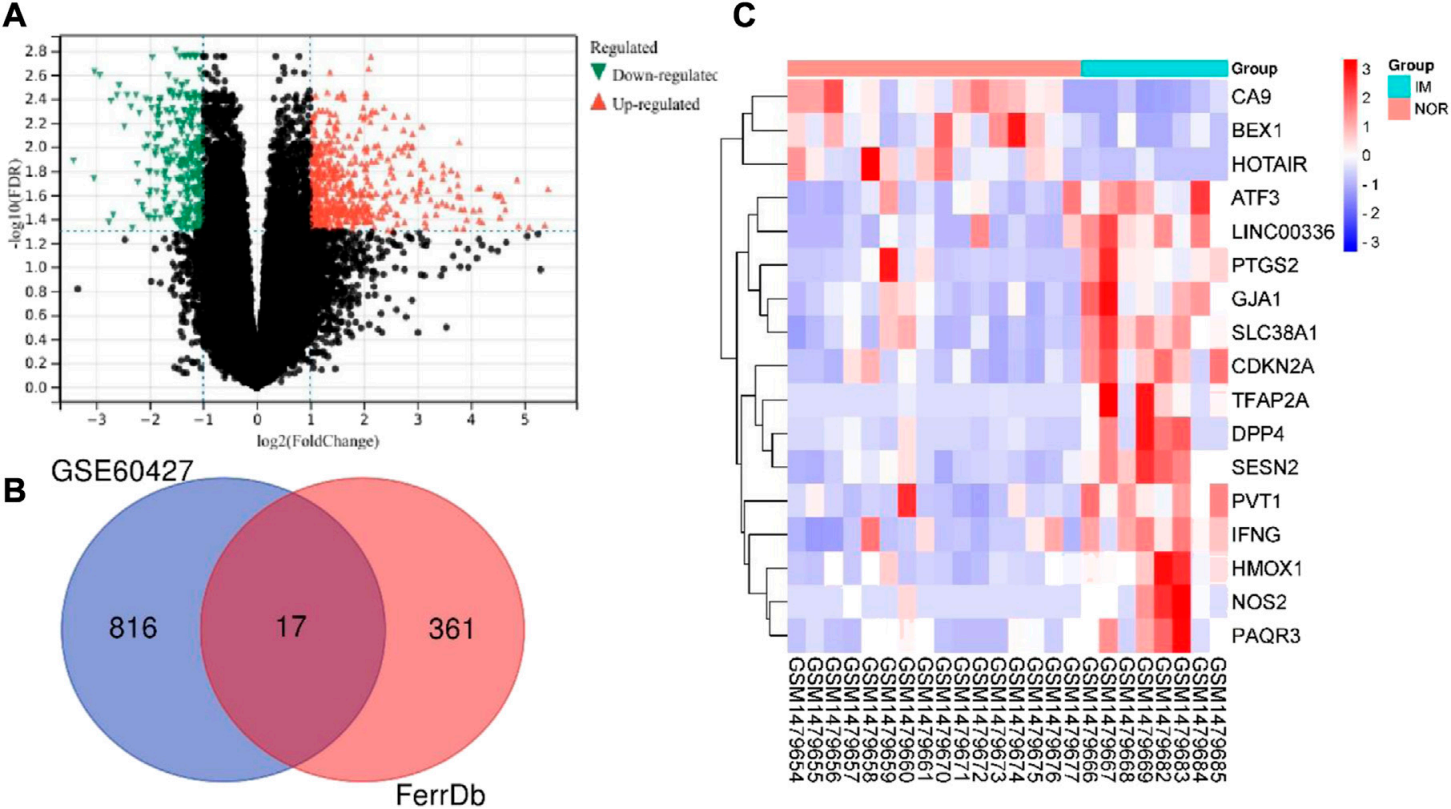
Identification differentially expressed ferroptosis-associated genes. **(A)** Volcano plot of DEGs of GSE60427 (red: upregulated DEGs. green: downregulated DEGs.) **(B)** After the intersection, 17 DEFRGs were identified based on the Venn diagram. **(C)**Heatmap of final DEFRGs (red: high expression; blue: low expression).

The total mRNA of GSE60427 was 46,204. The “limma” package in R software was used to analyze the differential expression of IM and normal controls. According to the predetermined threshold (|log2FC| ≥ 1 and adjusted *p* < 0.05), we identified 833 DEGs from GSE60427 (Figure 2A), including 517 upregulated genes and 316 downregulated genes. In order to identify DEGs related to ferroptosis, 378 data sets of ferroptosis-related gene were obtained from FerrDb database. Finally, 17 DEFRGs (Figure 2B) were selected according to Venn diagram. The clustering heat map shows the expression pattern of DEFRGs between samples (Figure 2C).

### 3.2 Functional enrichment analysis

**FIGURE 3:**
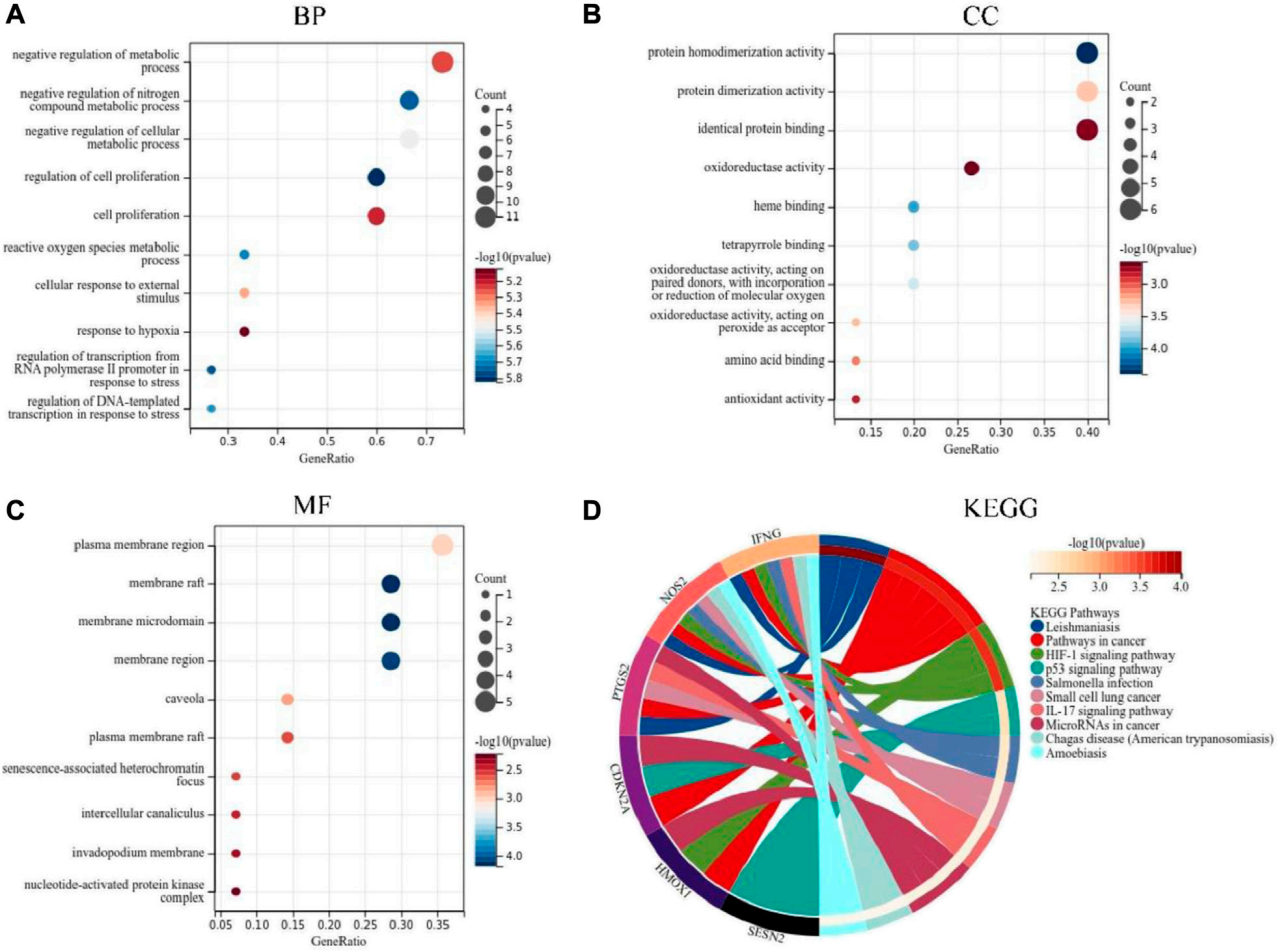
GO and KEGG pathway enrichment analyses of DEFRGs. **(A)** Top 10 GO (gene ontology) biological processes pathway. **(B)** Top 10 GO cellular component pathway. **(C)** Top 10 GO molecular function pathway. **(D)** Top 10 KEGG pathway.

In order to further understand the function of DEFRGs, GO and KEGG Pathway enrichment analysis were performed. Go analysis showed that the biological process (BP) was mainly concentrated in: regulation of cell proliferation, regulation of transcription from RNA polymerase II promoter in response to stress, negative regulation of nitrogen compound metabolic process, reactive oxygen species metabolic process, regulation of DNA-emplated transcription in response to stress, negative regulation of cellular metabolic process, cellular response to external stimulus, negative regulation of metabolic process, cell proliferation, response to hypoxia (Figure 3A). The main CC included: membrane raft, membrane raft, membrane microdomain, membrane region, plasma membrane region, caveola, plasma membrane raft, senescence-associated heterochromatin focus, intercellular canaliculus, invadopodium membrane, nucleotide-activated protein kinase complex (Figure 3B). The main MF included: protein homodimerization activity, heme binding, tetrapyrrole binding, oxidoreductase activity, acting on paired donors, with incorporation or reduction of molecular oxygen, protein dimerization activity, oxidoreductase activity, acting on peroxide as acceptor, amino acid binding, antioxidant activity, identical protein binding and oxidoreductase activity (Figure 3C). The KEGG pathways were mainly concentrated in: Leishmaniasis, Pathways in cancer, HIF-1 signaling pathway, p53 signaling pathway, *Salmonella* infection, Small cell lung cancer, IL-17 signaling pathway, MicroRNAs in cancer, Chagas disease (American trypanosomiasis) and Amoebiasis (Figure 3D).

### 3.3 Identification of key DEFRGs

**FIGURE 4:**
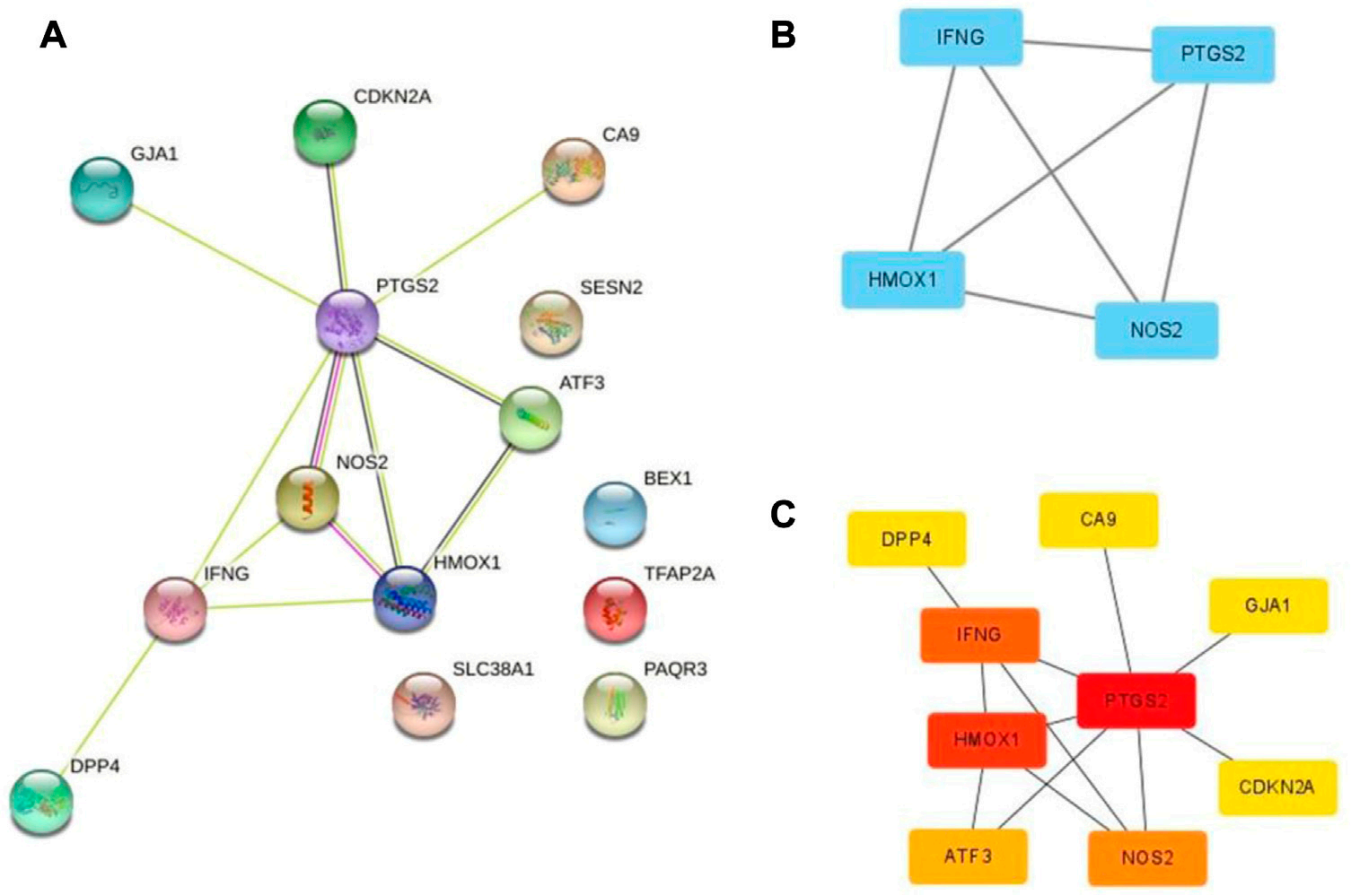
PPI network of ferroptosis DEFRGs. **(A)** The PPI among 17 differentially expressed ferroptosis-related genes. **(B)** Key module of the PPI network screened by MCODE plugin. **(C)** Hub genes screened by cytoHubba plugin.

To determine the interaction between DEFRGs, we used the STRING database to build a PPI network with 14 nodes and 12 edges (Figure 4A). And Cytoscape was used for the subsequent analysis of PPI network. The MCODE plug-in identified a key module (Figure 4B) with 4 nodes and 6 edges, including PTGS2, HMOX1, IFNG, and NOS2. Then nine central genes (Figure 4C) were analyzed and identified by MCC algorithm in cytoHubba plug-in, including PTGS2, HMOX1, IFNG, NOS2, ATF3, CDKN2A, DPP4, CA9, GJA1. The overlapping genes were considered as hub genes: PTGS2, HMOX1, IFNG, and NOS2.

### 3.4 Performance of hub genes to diagnose IM

**FIGURE 5:**
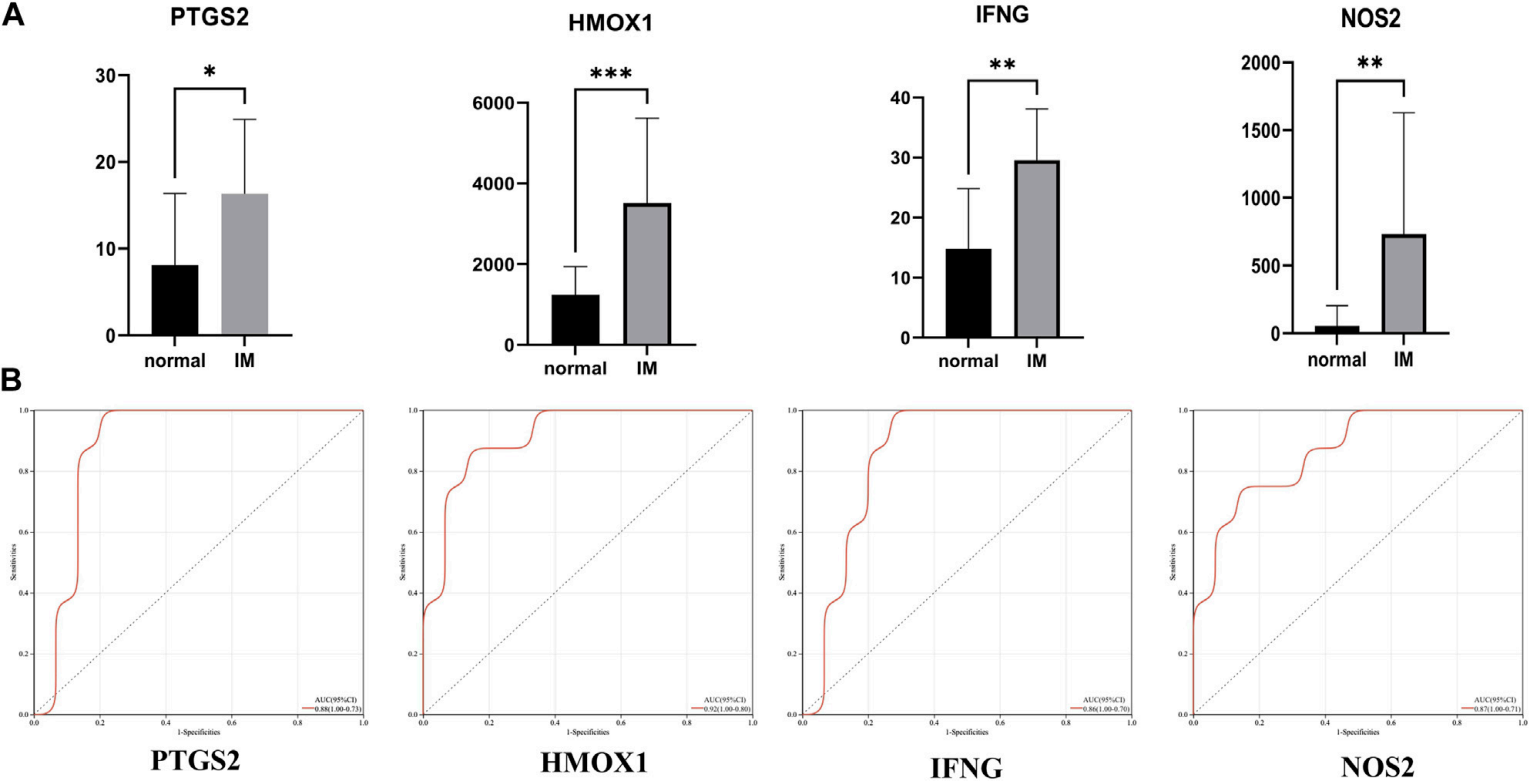
**(A)** Relative expression of PTGS2, HMOX1, IFNG, and NOS2 in GSE60427 dataset. **(B)** The receiver operating characteristic (ROC) curve of PTGS2, HMOX1, IFNG and NOS2 in GSE60427 dataset. *p < 0.05; **p < 0.01; ***p < 0.001.

In the training set (GSE60427), four hub genes related to ferroptosis are significantly overexpressed in IM patients compared with normal patients (Figure 5A). Figure 5B shows the receiver operating characteristic (ROC) curve of PTGS2, HMOX1, IFNG and NOS2 diagnosis of IM. The area under the ROC curve of IM diagnosed by PTGS2 is 0.88 (95% CI 0.73–1.0), the area under ROC curve of IM diagnosed by HMOX1 is 0.92 (95% CI 0.80–1.0), the area under ROC curve of IFNG diagnosis of IM is 0.86 (95% CI 0.70–1.0), and the area under ROC curve of NOS2 diagnosis of IM is 0.87 (95% CI 0.71–1.0).

**FIGURE 6:**
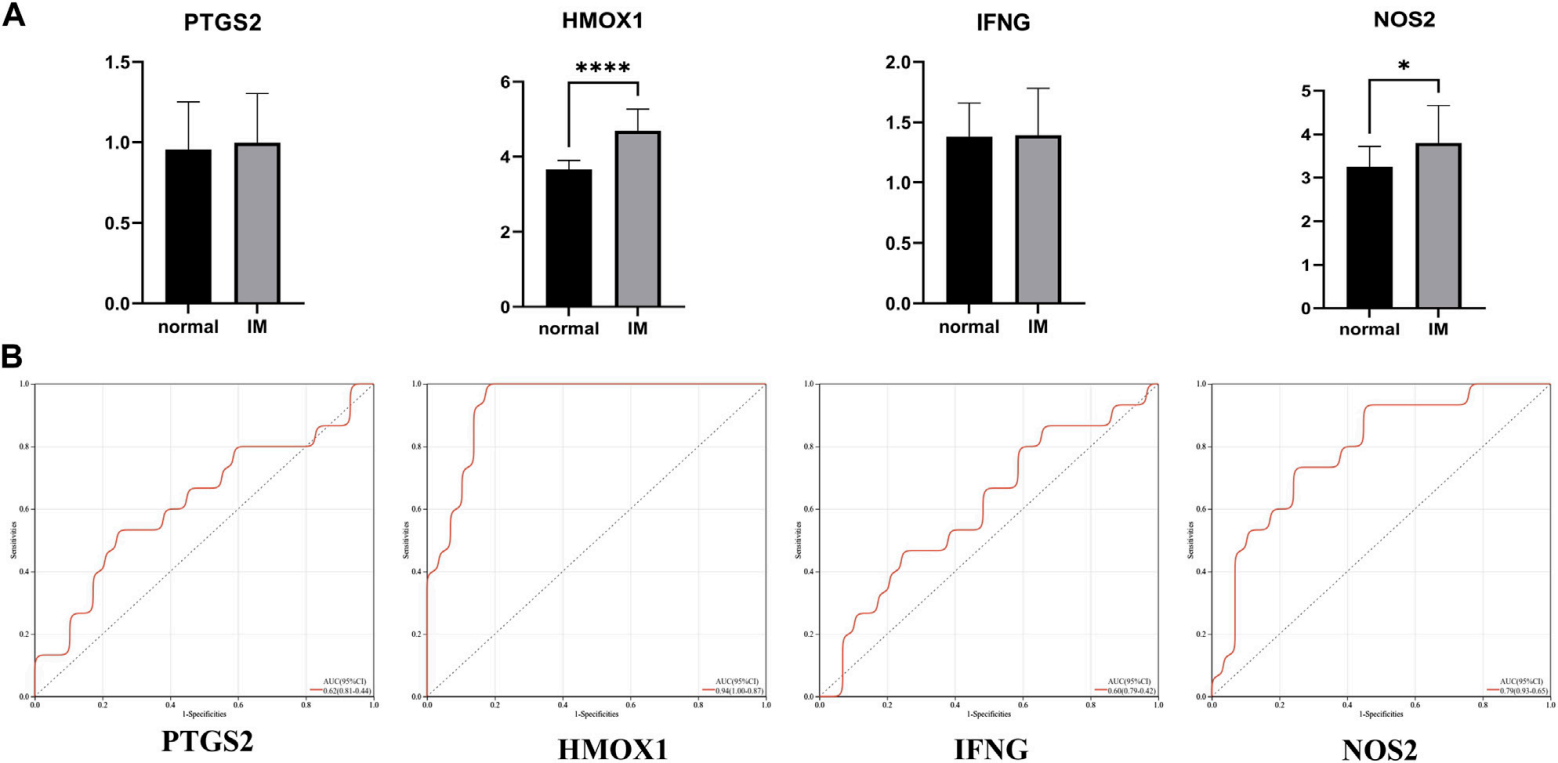
**(A)** Relative expression of PTGS2, HMOX1, IFNG, and NOS2 in GSE78523 dataset. **(B)** The receiver operating characteristic (ROC) curve of PTGS2, HMOX1, IFNG, and NOS2 in GSE78523 dataset. *p < 0.05; ****p < 0.0001.

The verification set (GSE78523) included 15 normal subjects and 30 IM patients, which were used to verify the above four hub genes. The results show that the differential expression of HMOX1 and NOS2 in GSE78523 dataset is statistically significant (Figure 6A). The areas under the ROC curve of PTGS2, HMOX1, IFNG, and NOS2 are 0.62, 0.94, 0.60, 0.79, respectively (Figure 6B). The results of external verification dataset show the validity and robustness of HMOX1 and NOS2 genes.

### 3.5 qRT-PCR experiment

**FIGURE 7:**
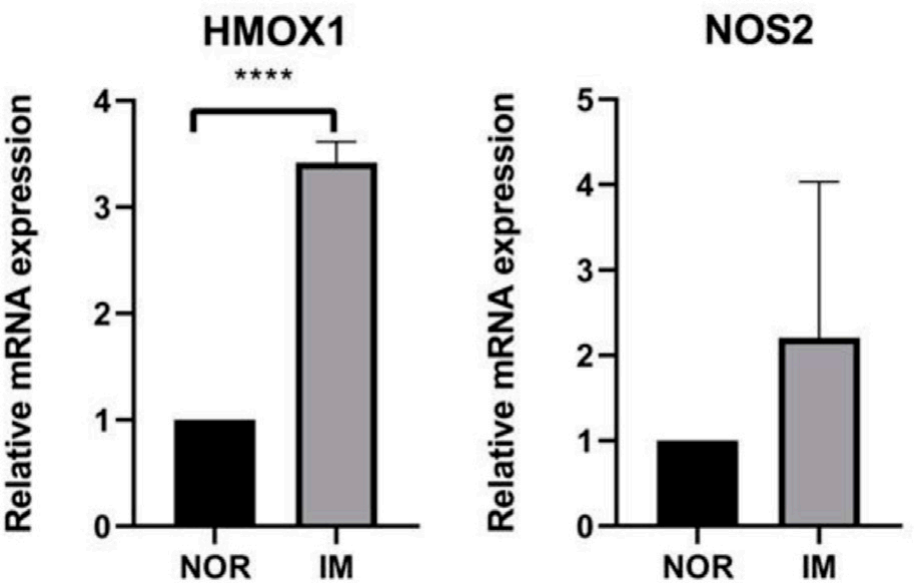
The expression levels of the three FRGs between healthy control group (*n* = 5) and IM group (*n* = 5). ****p < 0.0001.

In order to further confirm the results of bioinformatics analysis, we collected 5 normal gastric mucosa samples and 5 IM gastric mucosa samples. Figure 7 shows that there are significant changes in the expression levels of HMOX1 in normal gastric mucosa and IM gastric mucosa (*p* < 0.05).

### 3.6 Survival analysis of HMOX1 gene

**FIGURE 8:**
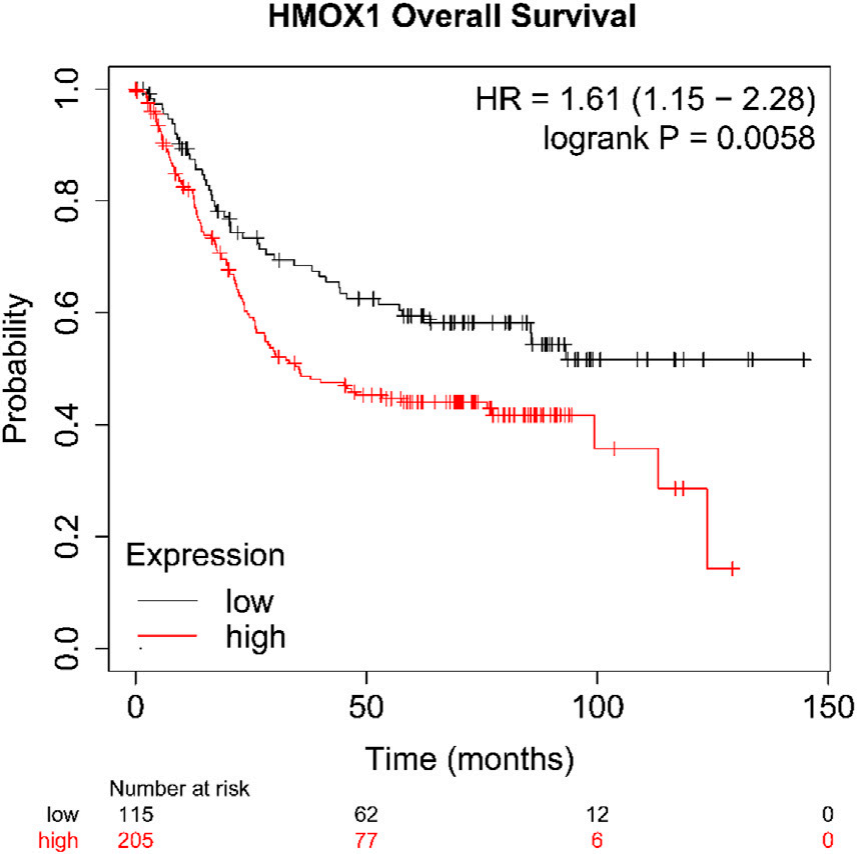
The survival analysis of HMOX1 (*p* < 0.05; HR = 1.61).

As we described earlier, IM is a key link in gastric precancerous lesions, so we have reason to suspect that HMOX1 also plays a key role in gastric cancer. In order to verify our conjecture, we used Kaplan-Meier Plotter database to explore the effects of high and low expression of HMOX1 gene on overall survival (OS) of patients with gastric cancer. The results (Figure 8) shows that the patients with high expression levels of HMOX1 gene have shorter OS (*p* < 0.05).

### 3.7 Immune infiltration landscapes

**FIGURE 9:**
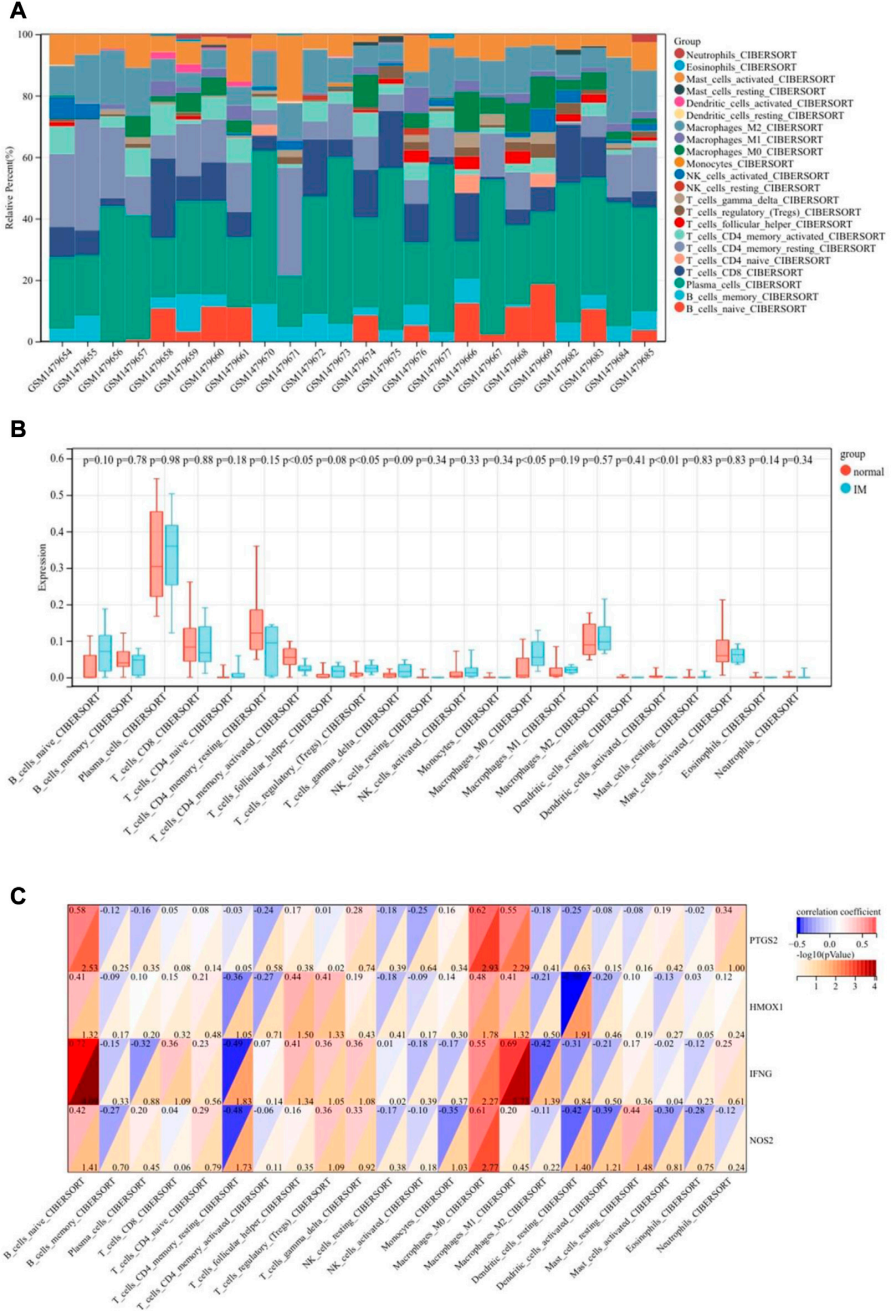
Immune landscape analysis. **(A)** Bar charts of 22 immune cell proportions. **(B)** Differential immune cell infiltration between IM and control groups. **(C)** Correlation between differentially infiltrated immune cells and hub genes.

We used the CIBERSORT algorithm to analyze the difference of immune cells between the IM group and the control group. Figures 9A, B shows 22 types of infiltrating immune cells. Compared with the control group, the proportion of T cells regulatory (Tregs) and macrophages M0 in the IM group are relatively high. On the contrary, T cells CD4 memory activated and dendritic cells activated are significantly lower in IM. The correlation between central gene expression and differentially infiltrated immune cells is shown in Figure 9C, in which B cells naive has a significantly positive correlation with PTGS2 (r = 0.58) and IFNG (r = 0.72). T cells CD4 memory resting is negatively correlated with IFNG (r = 0.49) and NOS2 (r = 0.48). Macrophages M0 is positively correlated with PTGS2 and NOS2, and positively correlated with IFNG. Macrophages M1 has a significantly positive correlation with IFNG and a positive correlation with PTGS2. Dendritic cells resting is negatively correlated with HMOX1 and NOS2.

## 4 Discussion

Although IM is considered as an important inducer for the development of intestinal GC (Kinoshita et al., 2017), the specific mechanism of the occurrence and development of IM is not clear. The occurrence of IM involves cell structural degeneration, activation of progenitor cell-related genes and re-entry into the cell cycle (Willet et al., 2018). Most people’s IM occurs in the context of chronic gastritis. H.pylori (HP) infection is generally considered to be a major influencing factor, which activates immune cells and causes DNA damage and elevated levels of reactive oxygen species (ROS) (Kang et al., 2022). Ferroptosis is a new form of non-apoptotic cell death, which is iron-dependent and non-apoptotic cell death, characterized by the accumulation of lipid-based ROS. Several studies (Sun et al., 2020; Shao et al., 2021; Zhao et al., 2021) have proved that FRGs play a key role in the occurrence and development of gastric cancer and can be used to predict the prognosis and clinical status of gastric cancer patients. However, the research on the role of ferroptosis in precancerous lesions of gastric cancer, especially in IM is still blank.

This study explored the molecular characteristics of ferroptosis associated with IM by analyzing the DEGs of IM cases compared with healthy subjects. A total of 17 DEFRGs were obtained from GSE60427 and FerrDb dataset. In addition, the GO terms and pathway of the 17 DEFRGs were studied by GO and KEGG analysis. GO analysis showed that these DEFRGs were significantly enriched in reactive oxygen species metabolic process response to hypoxia and oxidoreductase activity. It is suggested that these DEFRGs are related to reactive oxygen species. Through KEGG Pathway enrichment analysis, we observed that these genes were enriched in Pathways in cancer, HIF-1 signaling pathway, p53 signaling pathway and other signal pathways. HIF1A (Hypoxia Inducible Factor 1 Subunit Alpha) is a transcription factor that mediates homeostatic responses to reduced oxygen availability in the microenvironment. Studies have shown that (Yang et al., 2019) the expression of HIF1A can limit the occurrence of ferroptosis. Ferroptosis induced by targeting HIF1A in osteoclasts may be a new method for the treatment of osteoporosis (Ni et al., 2021). P53 is the most widely studied tumor suppressor gene and is considered to be involved in the occurrence of cancer (Levine, 2020). Many studies have shown that p53 is closely related to ferroptosis (Gnanapradeepan et al., 2018). P53 has been demonstrated to promote cancer ferroptosis predominantly via regulating SLC7A11 expression and cystine uptake (Jiang et al., 2015). In addition, p53 gene mutation is an important initiating factor in the occurrence and development of GC (Lei et al., 2022). In the progression of gastric precancerous lesions, the expression level of p53 increases gradually with the progress of the disease from normal mucosa. P53 mutation is a key event in the transition from IM to GC (Busuttil et al., 2014). The above results are helpful to describe the pathways related to ferroptosis and IM, which may increase our understanding of the IM mechanism.

After that, we analyzed 17 DEFRGs-built PPI networks by Cytoscape. MCODE and cytoHubba plug-ins screened out a key clustering module, and four genes (PTGS2, HMOX1, IFNG, NOS2) may be closely related to the occurrence of IM. Among them, the area under ROC curve and differential expression of HMOX1 and NOS2 genes in training and verification set are significant. HMOX1 is an essential enzyme in heme catabolism. The carbon monoxide (CO) produced by it can protect the damaged gastric mucosa (Bakalarz et al., 2021). In gastric cancer, low HMOX1 expression promotes gastric cancer cell apoptosis, inhibits proliferation and invasion, and correlates with increased overall survival in gastric cancer patients (Ren et al., 2017). NOS2 encodes nitric oxide synthase. NO plays an important role in normal gastric function by controlling gastric blood flow and maintaining the integrity of gastric mucosal barrier (Magierowski et al., 2015). The change of NO formation is related to the occurrence and progression of gastric cancer. NO can induce DNA damage by inhibiting DNA repair activity or directly modifying DNA structure (Tamir et al., 1996). Exposure to exogenous NO donors or increased expression of NOS2 can lead to the accumulation of p53 mutation (Forrester et al., 1996). At the same time, p53 can also negatively regulate NOS2 expression. The loss of p53 function has been shown to increase the expression of HMOX1 and help to increase tumor growth (Wada et al., 2015). In gastric cancer, the increase of NOS2 expression correlates with the decrease of survival rate (Zhang et al., 2011) and disease stage (Wang et al., 2005).What’s more, the expression of NOS2 is associated with metastasis of gastric cancer and the increase of angiogenesis (de Oliveira et al., 2017). These results suggest that HMOX1 and NOS2 play an important role in the formation of IM.

The induction of the innate immune response of gastric epithelial cells and myeloid cells by HP effectors plays a critical role in the outcome of the infection (Gobert and Wilson, 2022). However, no research has focused on the role of immune microenvironment in IM. Therefore, we studied the infiltration of immune cells in IM by CIBERSORT algorithm. The results showed that some immune cells in IM were significantly different from those in normal gastric tissue. Compared with normal stomach, the proportion of T cells regulatory (Tregs) and macrophages M0 was relatively higher, while the proportion of T cells CD4 memory activated and dendritic cells activated was lower. At the same time, there was a correlation between hub gene expression and differentiated infiltrating immune cells. However, the exact mechanism of their interaction remains to be further studied.

As far as we know, this is the first study to focus on the role of ferroptosis in IM. However, this study still has its limitations. The data we analyzed were downloaded from the GEO dataset, and further prospective clinical studies are necessary to validate the observations. For now, our study can provide a theoretical basis for further exploration of ferroptosis-related phenotypes in IM studies.

## 5 Conclusion

In this study, we identified four bub genes (PTGS2, HMOX1, IFNG, NOS2) related to ferroptosis in IM. Among them, HMOX1 has diagnostic value and may be biomarkers and therapeutic targets for the diagnosis of IM. This study may help to understand the pathogenesis of IM and to study the best treatment strategy for IM patients.

## Data Availability

Publicly available datasets were analyzed in this study. This data can be found here: Gene Expression Omnibus database (http://www.ncbi.nlm.nih.gov/geo/): GSE60427 and GSE78523; FerrDb database (http://www.zhounan.org/ferrdb); Kaplan-Meier Plotter database (http://kmplot.com/analysis/).
